# A Genome-Wide Association Study of Optic Disc Parameters

**DOI:** 10.1371/journal.pgen.1000978

**Published:** 2010-06-10

**Authors:** Wishal D. Ramdas, Leonieke M. E. van Koolwijk, M. Kamran Ikram, Nomdo M. Jansonius, Paulus T. V. M. de Jong, Arthur A. B. Bergen, Aaron Isaacs, Najaf Amin, Yurii S. Aulchenko, Roger C. W. Wolfs, Albert Hofman, Fernando Rivadeneira, Ben A. Oostra, Andre G. Uitterlinden, Pirro Hysi, Christopher J. Hammond, Hans G. Lemij, Johannes R. Vingerling, Caroline C. W. Klaver, Cornelia M. van Duijn

**Affiliations:** 1 Department of Epidemiology, Erasmus Medical Center, Rotterdam, The Netherlands; 2 Department of Ophthalmology, Erasmus Medical Center, Rotterdam, The Netherlands; 3 Glaucoma Service, The Rotterdam Eye Hospital, Rotterdam, The Netherlands; 4 Department of Neurology, Erasmus Medical Center, Rotterdam, The Netherlands; 5 Department of Ophthalmology, University Medical Center Groningen, University of Groningen, Groningen, The Netherlands; 6 Department of Ophthalmogenetics, The Netherlands Institute for Neuroscience, Royal Netherlands Academy of Arts and Sciences, Amsterdam, The Netherlands; 7 Department of Ophthalmology, Academic Medical Center, Amsterdam, The Netherlands; 8 Department of Clinical Genetics, Academic Medical Center, Amsterdam, The Netherlands; 9 Department of Internal Medicine, Erasmus Medical Center, Rotterdam, The Netherlands; 10 Department of Clinical Genetics, Erasmus Medical Center, Rotterdam, The Netherlands; 11 Department of Twin Research and Genetic Epidemiology, King's College London, London, United Kingdom; Stanford University School of Medicine, United States of America

## Abstract

The optic nerve head is involved in many ophthalmic disorders, including common diseases such as myopia and open-angle glaucoma. Two of the most important parameters are the size of the optic disc area and the vertical cup-disc ratio (VCDR). Both are highly heritable but genetically largely undetermined. We performed a meta-analysis of genome-wide association (GWA) data to identify genetic variants associated with optic disc area and VCDR. The gene discovery included 7,360 unrelated individuals from the population-based Rotterdam Study I and Rotterdam Study II cohorts. These cohorts revealed two genome-wide significant loci for optic disc area, rs1192415 on chromosome 1p22 (p = 6.72×10^−19^) within 117 kb of the *CDC7* gene and rs1900004 on chromosome 10q21.3-q22.1 (p = 2.67×10^−33^) within 10 kb of the *ATOH7* gene. They revealed two genome-wide significant loci for VCDR, rs1063192 on chromosome 9p21 (p = 6.15×10^−11^) in the *CDKN2B* gene and rs10483727 on chromosome 14q22.3-q23 (p = 2.93×10^−10^) within 40 kbp of the *SIX1* gene. Findings were replicated in two independent Dutch cohorts (Rotterdam Study III and Erasmus Rucphen Family study; N = 3,612), and the TwinsUK cohort (N = 843). Meta-analysis with the replication cohorts confirmed the four loci and revealed a third locus at 16q12.1 associated with optic disc area, and four other loci at 11q13, 13q13, 17q23 (borderline significant), and 22q12.1 for VCDR. *ATOH7* was also associated with VCDR independent of optic disc area. Three of the loci were marginally associated with open-angle glaucoma. The protein pathways in which the loci of optic disc area are involved overlap with those identified for VCDR, suggesting a common genetic origin.

## Author Summary

Morphologic characteristics of the optic nerve head are involved in many ophthalmic diseases. Its size, called the optic disc area, is an important measure and has been associated with e.g. myopia and open-angle glaucoma (OAG). Another important and clinical parameter of the optic disc is the vertical cup-disc ratio (VCDR). Although studies have shown a high heritability of optic disc area and VCDR, its genetic determinants are still undetermined. We therefore conducted a genome-wide association (GWA) study on these quantitative traits, using data of over 11,000 Caucasian participants, and related the findings to myopia and OAG. We found evidence for association of three loci with optic disc area: *CDC7*/*TGFBR3* region, *ATOH7*, and *SALL1*; and six with VCDR: *CDKN2B*, *SIX1*, *SCYL1*, *CHEK2*, *ATOH7*, and *DCLK1*; and additionally one borderline significant locus: *BCAS3*. None of the loci could be related to myopia. There was marginal evidence for association of *ATOH7*, *CDKN2B*, and *SIX1* with OAG, which remains to be confirmed. The present study reveals new insights into the physiological development of the optic nerve and may shed light on the pathophysiological protein pathways leading to (neuro-) ophthalmologic diseases such as OAG.

## Introduction

The optic nerve head, or optic disc, is the place where the axons of the retinal ganglion cells leave the eye and form the optic nerve. Its morphology, visible by ophthalmoscopy, is important in the diagnosis and follow-up of patients with (neuro-) ophthalmologic diseases, such as ischemic and hereditary optic neuropathies, optic neuritis, papilledema and primary open-angle glaucoma (OAG). Optic disc parameters of interest are the surface of the optic nerve head referred to as the optic disc area (measured in units of mm^2^), and the vertical cup-disc ratio (VCDR). The optic disc area is associated with general characteristics (such as body height) as well as ocular ones (such as axial length) [1], [2]. The relation to axial length makes the optic disc size directly relevant for nearsightedness (myopia), one of the most common ophthalmic disorders. Furthermore, it has been suggested that larger optic discs may suffer more from intraocular pressure-related stress, a strong risk factor for OAG [3]. However, the association of the size of the optic disc to OAG is not clear since it has been argued that larger optic discs may have a larger anatomical reserve for various optic neuropathies such as OAG due to higher number of nerve fibers [4]. Effects may even partially counteract each other [4].

The VCDR is a parameter commonly used in the clinical glaucoma management [5]. The VCDR is determined by comparing (in a vertical direction) the size of the cup, a region without axons, to the size of the optic disc. An increase in VCDR may indicate the occurrence of glaucomatous changes of the optic nerve head, referred to as glaucomatous optic neuropathy [6]. In addition, an unusual large VCDR at a single observation is a significant determinant of glaucoma [7], [8]. The heritability of the optic disc area and VCDR are estimated to be around 52–59% and 48–80%, respectively, [9]–[12] suggesting a major role for genetic factors. This prompted us to study the genes determining the optic disc area and VCDR as endophenotypes for myopia and OAG.

To identify genetic determinants of optic disc area and VCDR, we performed a genome-wide association study (GWAS) of optic disc area and VCDR using data from Caucasian participants of the Rotterdam Study [RS] (cohort I and II, in which participants have an identical age distribution and eye assessment) and replicated our findings in three independent cohorts of Caucasian ethnicity: the Rotterdam Study III [RS-III, a younger cohort], the Erasmus Rucphen Family [ERF] study and the TwinsUK cohort (see Materials and Methods for details of all cohorts). Next, we examined whether the genome-wide significant Single Nucleotide Polymorphisms (SNPs) were related to myopia and OAG using data from patients with (one of) these diseases from the Rotterdam Study I.

## Results

### Study samples

The discovery cohorts included 5,312 (RS-I) and 2,048 (RS-II) participants who were genotyped and had reliable optic disc data, resulting in a total of 7,360 participants included in the primary GWAS discovery set. A small fraction (205 from RS-I and 90 from RS-II), had missing or unreliable baseline data; for these we used the data available at follow-up. From RS-III, 1,966 participants were included, and from ERF 1,646, resulting in a total of 10,972 when combining the discovery and replication cohorts from the Netherlands, and 11,815 when also including the 843 participants of TwinsUK. Table 1 summarizes the general characteristics of the discovery and replication cohorts. There are significant differences between the cohorts in terms of age (discovery cohort is older), gender (TwinsUK includes only women) and optic disc parameters (due to different disc-assessment techniques [see Materials and Methods]; the analyses were adjusted for this difference).

**Table 1:** Characteristics of the five study populations presented as mean ± standard deviation (range) unless stated otherwise.

	RS-I/RS-II	RS-III	ERF	TwinsUK
Total sample size (N)	7,360	1,966	1,646	843
Age (years)	67.0±8.4 (55–99)	55.6±5.5 (45–89)	46.8±14.1 (18–84)	56.1±12.7 (16–83)
Gender, N(%) female	4,208 (57.2)	1,102 (56.1)	942 (57.2)	818 (97.0)
Disc area (mm^2^)*	2.40±0.48 (0.58–6.20)	1.92±0.45 (0.70–7.20)	1.92±0.37 (1.07–4.33)	2.59±0.65 (0.75–6.96)
Vertical cup-disc ratio*	0.50±0.14 (0.00–0.89)	0.42±0.17 (0.00–1.00)	0.46±0.15 (0.00–0.84)	0.32±0.10 (0.07–0.70)

*In RS-I, RS-II and TwinsUK measured with stereoscopic images, in RS-III and ERF with confocal scanning laser ophthalmoscopy.

Figure S1 and Figure S2 show the Q-Q plots for the observed versus expected p-values for each individual study and for the meta-analysis of the discovery and replication cohorts for optic disc area and VCDR, respectively. Genomic control for all four cohorts showed low dispersion for optic disc area as well as for VCDR with inflation factors in the range of 1.024 and 1.061.

### Optic disc area

**Figure 1:**
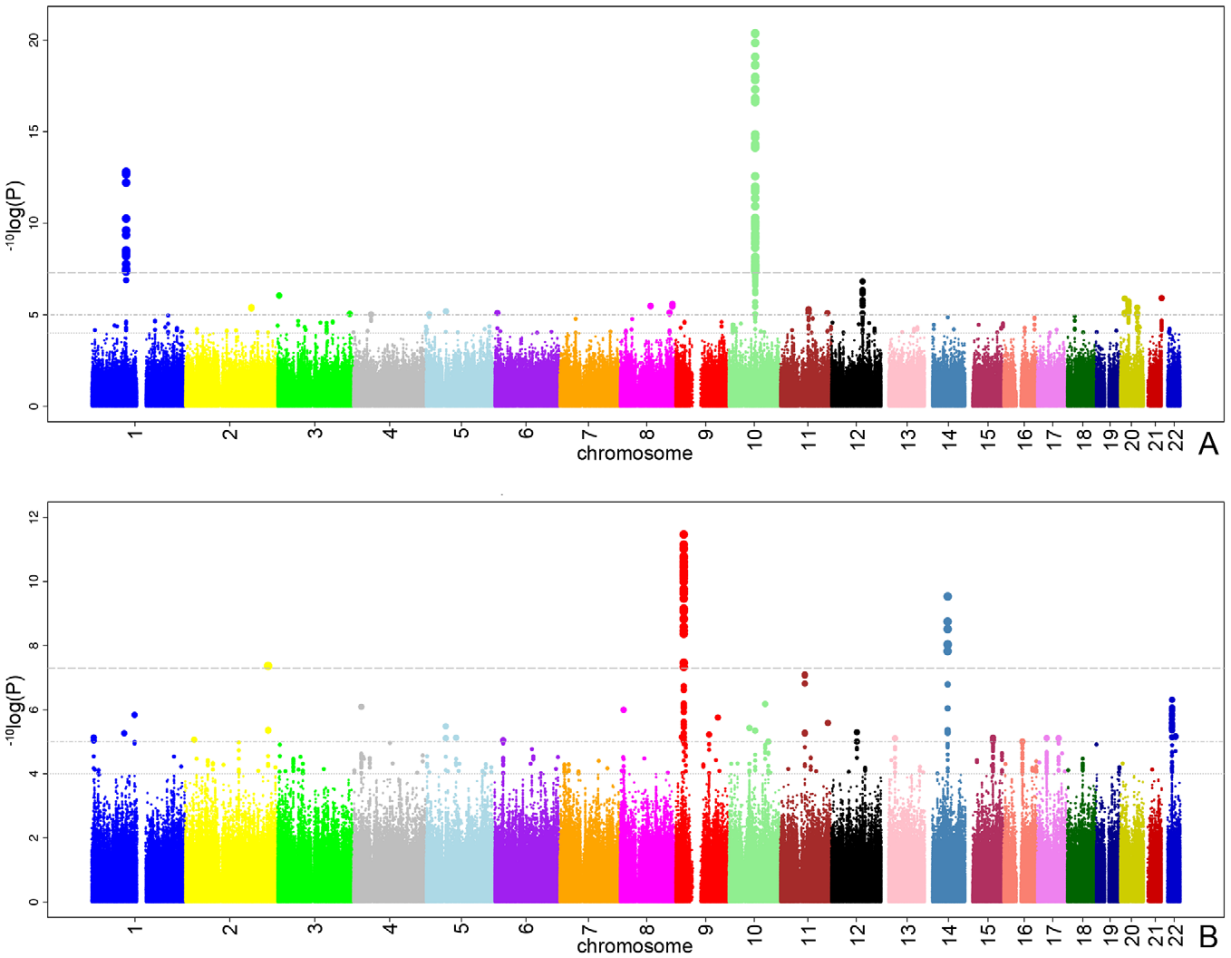
The ^−10^log p-plots for the meta-analyzed RS-I/RS-II genome-wide association study. Plot (A) of disc area and plot (B) of vertical cup-disc ratio. The upper line represents the genome-wide significance threshold: p = 5×10^−8^. The middle and bottom line represents the 10^−5^ and 10^−4^ respectively.

Figure 1A presents the ^−10^log p-plot for the primary discovery cohort for optic disc area and shows two loci on chromosomes 1 and 10, including 192 SNPs that are beyond the genome-wide significance threshold of 5×10^−8^. Exclusion of OAG (N = 188) and myopia (N = 115) cases did not alter the results. Replication analyses in two independent cohorts of Dutch origin (RS-III and ERF study) showed that the findings from all cohorts were consistent in the direction of the effect with p-values ranging from 1.69×10^−3^ to 2.39×10^−10^ (Table 2). The combined analysis of the discovery and Dutch replication cohorts yielded an overall p-value 1.82×10^−27^ for rs1192415 (optic disc area increased by 0.064±0.006 mm^2^ [beta ± standard error] when comparing those heterozygous with homozygous persons for the reference allele), and p-value 2.05×10^−32^ for rs1900004 (optic disc area decreased by 0.068±0.006 mm^2^). Table 2 shows the results of the top SNPs of all loci with p-values <10^−6^ observed in the meta-analysis. The meta-analysis of the four Dutch cohorts revealed a cluster of 10 SNPs on chromosome 16q12.1 showing borderline genome-wide significant evidence for association with the optic disc area (p = 6.48×10^−8^). When joining the Dutch data with the TwinsUK series (Table 3), this region became genome-wide significant (p = 5.07×10^−9^). Table 3 shows that also the chromosome 1 and 10 regions were also replicated consistently in the TwinsUK cohort.

**Table 2:** Results of top SNPs of all associated loci with p-value <10^−6^ on disc area in the meta-analysis for each individual cohort and the meta-analysis itself (results are presented as the effects per minor allele).

SNP	Chromosome location	Position	MA	RS-I/RS-II				RS-III				ERF				Meta-analysis				Name	Distance (b)	Number of SNPs on loci with p-value <10−6
				MAF	Beta	SE	P-value	MAF	Beta	SE	P-value	MAF	Beta	SE	P-value	MAF	Beta	SE	P-value			
rs1900004	10q21.3-q22.1	69670887	T	0.22	−0.114	0.009	2.67×10^−33^ *	0.23	−0.082	0.017	1.85×10^−6^	0.21	−0.033	0.008	5.28×10^−5^	0.22	−0.068	0.006	2.05×10^−32^ *	ATOH7/PBLD	9021	175
rs1192415	1p22	91849685	G	0.18	0.091	0.010	6.72×10^−19^ *	0.18	0.059	0.019	1.69×10^−3^	0.25	0.049	0.008	2.39×10^−10^ *	0.22	0.064	0.006	1.82×10^−27^ *	CDC7/TGFBR3	116719	61
rs1362756	16q12.1	50015791	C	0.29	0.036	0.009	4.85×10^−5^	0.28	0.032	0.016	4.92×10^−2^	0.27	0.023	0.007	1.56×10^−3^	0.28	0.028	0.005	6.48×10^−8^	SALL1	1154095	10

*Significant at a p-value of 5×10^−8^; SNP = single nucleotide polymorphism; MA(F) = minor allele (frequency); SE = standard error.

**Table 3:** Results of replication in the TwinsUK cohort of the three revealed loci on disc area with their meta-analyzed results of all five cohorts.

Most significant SNP	Minor allele	Minor allele frequency	Chromosome location	Position	delta disc area per allele (mm^2^)		P-value	delta disc area per allele in meta-analysis of all five cohorts (mm^2^)		P-value in meta-analysis of all five cohorts
Disc area					Beta	SE		Beta	SE	
rs1900004	T	0.24	10q21.3-q22.1	69670887	−0.133	0.038	4.64×10^−4^ *	−0.070	0.006	2.71×10^−35^ **
rs1192415	G	0.18	1p22	91849685	0.091	0.041	2.60×10^−2^ *	0.065	0.006	2.77×10^−28^ **
rs1362756	C	0.30	16q12.1	50015791	0.097	0.037	8.29×10^−3^ *	0.030	0.005	5.07×10^−9^ **

*Significant at a p-value of 0.05.

**Significant at a p-value of 5×10^−8^; SNP = single nucleotide polymorphism; SE = standard error.

**Figure 2:**
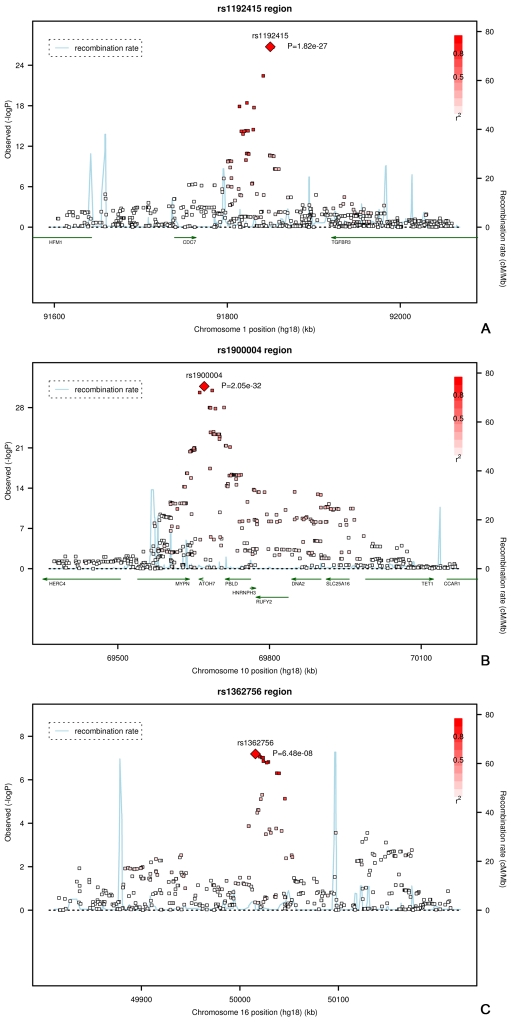
Regional plots of the three loci associated with optic disc area. Plots (A–C) show the loci on on chromosome 1, 10, and 16, respectively.

The regions of interest for optic disc area are shown in Figure 2. The first region on chromosome 1p22 is located between the cell division cycle 7 (*CDC7*) and the transforming growth factor beta receptor 3 (*TGFBR3*) gene, but the SNPs in the intergenic region were most significant. The genome-wide significant region on chromosome 10q21.3-q22.1 was quite large and included several genes. The region includes the Myopalladin (*MYPN*) gene, the heterogeneous nuclear ribonucleoprotein H3 (2H9) (*HNRNPH3*) gene, RUN and FYVE domain containing (*RUFY2*) gene, DNA replication helicase 2 homolog (yeast) (*DNA2*) gene, solute carrier family 25 (mitochondrial carrier; Graves disease autoantigen), member 16 (*SLC25A16*) gene. However, the most significant evidence was found in the region between the atonal homolog 7 (*ATOH7*) gene and the phenazine biosynthesis-like protein domain containing *(PBLD)* gene. The nearest gene in the third region on chromosome 16q12.1 was the sal-like 1 (*SALL1*) gene. Together, the three SNPs associated with optic disc area explained up to 2.7% of the variation in optic disc area.

Next, we evaluated the association of these loci with clinically relevant ophthalmic outcomes (myopia and OAG; Table S1). None of the optic disc area loci were associated with myopia-related outcomes (p-values ranging from 0.09 to 0.80). Of the three loci associated with optic disc area we found only the 10q21.3-q22.1 locus to be marginally associated with OAG (p = 0.04 for rs1900004).

### Vertical cup-disc ratio

**Figure 3:**
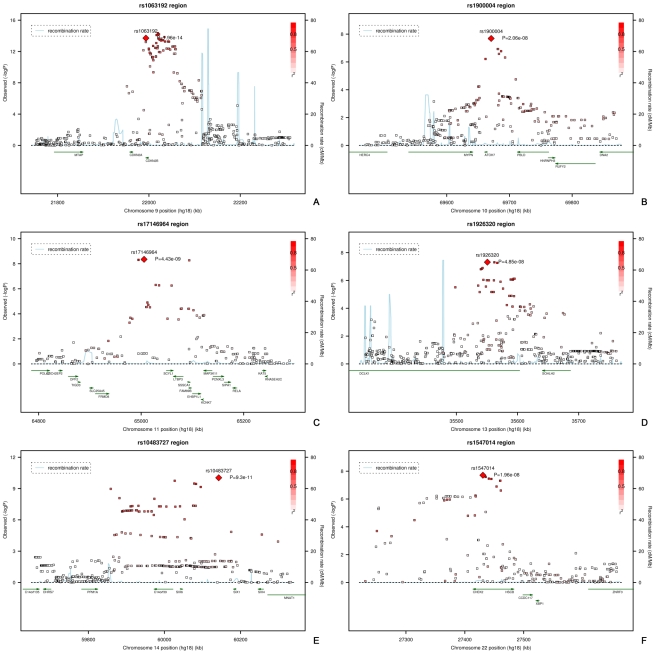
Regional plots of the six loci associated with vertical cup-disc ratio. Plots (A–F) show the loci on chromosome 9, 10, 11, 13, 14, and 22, respectively.

All analyses for VCDR were adjusted for optic disc area. Figure 1B presents the ^−10^log p-plot for the discovery cohorts (meta-analyzed RS-I/RS-II GWAS) for VCDR and shows two loci reaching genome-wide significance at a threshold of 5×10^−8^. Adjustment for the intraocular pressure did not alter the results nor did exclusion of the OAG cases. The combined analysis of the discovery and two Dutch replication cohorts yielded an overall p-value of 1.96×10^−14^ for rs1063192 and 9.30×10^−11^ for rs10483727 (Table 4). The regions of interest for VCDR are shown in Figure 3. The genome-wide significant region on chromosome 9 included two genes from the same gene family (cyclin-dependent kinase inhibitor 2A [*CDKN2A*] and *CDKN2B*). For chromosome 14, several genes were included in the region of interest. The strongest association was found for rs10483727 close to the sin oculis homeobox homolog 1 (*SIX1*) gene, but also several SNPs flanking *SIX6* were genome-wide significant as well as one SNP between RNA-binding motif 8B (*RBM8B*) and the protein phosphatase 1A (*PPM1A*) gene. Furthermore, there were four other loci that showed consistent evidence for association and reached genome-wide significance in the combined analysis of all Dutch cohorts (Table 4). This included the chromosome 10q21.3-q22.1 region identified for the optic disc area (Table 2). For chromosome 11q13, the most significant SNPs were found in between the FERM domain containing 8 (*FRMD8*) and the SCY1-like (*SCYL1*) gene. The region of interest also harboured latent transforming growth factor beta binding protein 3 (*LTBP3*). The genome-wide significant SNPs of these three regions were all in the same linkage disequilibrium block, hampering determination of the most important variant (Figure 3). Of the other two genome-wide significant loci, the SNPs point to the doublecortin–like kinase 1 (*DCLK1*) for chromosome 13q13, and CHK2 checkpoint homolog (*CHEK2*) for chromosome 22q12.1 (Figure 3).

**Table 4:** Results of top SNPs of all associated loci with p-value <10^−6^ on vertical cup-disc ratio in the meta-analysis for each individual cohort and the meta-analysis itself (results are presented as the effects per minor allele).

SNP	Chromosome location	Position	MA	RS-I/RS-II				RS-III				ERF				Meta-analysis				Name	Distance (b)	Number of SNPs on loci with p-value <10−6
				MAF	Beta	SE	P-value	MAF	Beta	SE	P-value	MAF	Beta	SE	P-value	MAF	Beta	SE	P-value			
rs1063192	9q21	21993367	G	0.45	−0.014	0.002	6.15×10^−11^*	0.46	−0.013	0.005	1.38×10^−2^	0.47	−0.015	0.005	2.54×10^−3^	0.46	−0.014	0.002	1.96×10^−14^*	CDKN2B	0	88
rs10483727	14q22-23	60142628	T	0.40	0.014	0.002	2.93×10^−10^*	0.39	0.001	0.005	7.81×10^−1^	0.45	0.014	0.005	4.95×10^−3^	0.41	0.012	0.002	9.30×10^−11^*	SIX1	39878	10
rs17146964	11q13	65005721	G	0.21	−0.014	0.003	7.94×10^−8^	0.21	−0.013	0.007	5.65×10^−2^	0.21	−0.010	0.006	1.05×10^−1^	0.21	−0.014	0.002	4.43×10^−9^*	SCYL1	43403	8
rs1547014	22q12.1	27430711	T	0.29	−0.011	0.002	7.20×10^−6^	0.30	−0.019	0.006	1.02×10^−3^	0.32	−0.010	0.005	7.34×10^−2^	0.29	−0.011	0.002	1.96×10^−8^*	CHEK2	0	29
rs1900004	10q21.3-q22.1	69670887	T	0.22	−0.012	0.003	4.49×10^−6^	0.23	−0.021	0.006	8.90×10^−4^	0.21	−0.007	0.006	2.98×10^−1^	0.22	−0.013	0.002	2.06×10^−8^*	ATOH7/PBLD	9021	10
rs1926320	13q13	35550617	C	0.24	0.011	0.003	1.45×10^−5^	0.25	0.020	0.006	1.29×10^−3^	0.27	0.008	0.006	1.41×10^−1^	0.24	0.012	0.002	4.85×10^−8^*	DCLK1	0	15
rs8068952	17q23	56641426	G	0.24	−0.012	0.003	7.85×10^−6^	0.24	−0.014	0.006	2.54×10^−2^	0.20	−0.007	0.006	2.47×10^−1^	0.23	−0.012	0.002	3.11×10^−7^	BCAS3	0	2
rs12025126	1p36.2-p36.1	8682141	C	0.28	−0.009	0.003	3.93×10^−4^	0.27	−0.011	0.006	6.62×10^−2^	0.32	−0.019	0.005	3.82×10^−4^	0.29	−0.011	0.002	4.14×10^−7^	RERE	0	5
rs2159128	19p13.3	901380	G	0.13	−0.016	0.005	3.16×10^−4^	0.14	−0.021	0.010	3.67×10^−2^	0.11	−0.032	0.011	2.45×10^−3^	0.13	−0.019	0.004	7.05×10^−7^	ARID3A	0	1

* = Significant at a p-value of 5×10^−8^; SNP = single nucleotide polymorphism; MA(F) = minor allele (frequency); SE = standard error.

Finally, when combining all top SNPs from the joint analysis of the four Dutch cohorts with the TwinsUK, one additional borderline genome-wide significant region emerged as genome-wide significant. The region comprises 2 SNPs on chromosome 17q23 (p = 2.81×10^−8^; Table 5). The combined effect of the six loci associated with VCDR explained 2.2% of the variation in the VCDR. Also for the VCDR none of the loci were associated to myopia at p<0.05. When we evaluated the association to OAG, four of the loci associated with VCDR were also found to be marginally associated with OAG, 9q21 (p = 0.017), 14q22-23 (p = 0.021), 11q13 (p = 0.049), and the overlapping gene *ATOH7* discussed earlier.

**Table 5:** Results of replication in the TwinsUK cohort of the three revealed loci on vertical cup-disc ratio with their meta-analyzed results of all five cohorts.

Most significant SNP	Minor allele	Minor allele frequency	Chromosome location	Position	delta disc area per allele (mm^2^)		P-value	delta disc area per allele in meta-analysis of all five cohorts (mm^2^)		P-value in meta-analysis of all five cohorts
rs1063192	G	0.44	9p21	21993367	−0.007	0.005	1.33×10^−1^	−0.013	0.002	4.35×10^−15^ **
rs10483727	T	0.44	14q22-23	60142628	0.012	0.005	1.36×10^−2^ *	0.012	0.002	1.01×10^−11^ **
rs17146964	G	0.20	11q13	65005721	−0.004	0.006	5.25×10^−1^	−0.012	0.002	3.72×10^−9^ **
rs1547014	T	0.26	22q12.1	27430711	−0.005	0.005	3.15×10^−1^	−0.011	0.002	1.48×10^−8^ **
rs1900004	T	0.24	10q21.3-q22.1	69670887	−0.005	0.006	3.83×10^−1^	−0.012	0.002	1.72×10^−8^ **
rs1926320	C	0.25	13q13	35550617	0.010	0.006	5.92×10^−2^	0.012	0.002	1.23×10^−8^ **
rs8068952	G	0.19	17q23	56641426	−0.018	0.007	6.69×10^−3^ *	−0.012	0.002	2.81×10^−8^ **
rs12025126	C	0.28	1p36.2-p36.1	8682141	−0.010	0.005	6.64×10^−2^	−0.011	0.002	5.69×10^−8^
rs2159128	G	0.08	19p13.3	901380	−0.012	0.010	2.41×10^−1^	−0.018	0.004	2.98×10^−7^

*Significant at a p-value of 0.05.

**Significant at a p-value of 5×10^−8^; SNP = single nucleotide polymorphism; SE = standard error.

## Discussion

In the present study we identified three genetic loci (10q21.3-q22.1, 1p22 and 16q12.1) associated with optic disc area, and six genetic loci (9q21, 14q22-23, 10q21.3-q22.1, 11q13, 13q13, and 22q12.1) associated with VCDR. Of these, one (10q21.3-q22.1) was associated with both quantitative traits. For these regions, the evidence for the association was genome-wide significant and our findings were consistently replicated in the independent replication cohorts. The SNPs in these loci were common variants with minor allele frequencies ranging from 0.21 to 0.46. The genome-wide significant SNPs of the present study were not in linkage disequilibrium with known missense mutations. The combined effect of the three SNPs involved in the optic disc area explained 2.7%, while the six loci associated with VCDR explained 2.2% of the variation.

The region with the strongest statistical evidence for association was a locus on chromosome 10q21.3-q22.1, which was associated with both optic disc area and VCDR, and included multiple genes. Although the genome-wide significant region is very large for the optic disc area analysis, the *ATOH7* gene (also known as *Math5*) showed the most significant evidence for association with VCDR. This gene is expressed in the retina where it controls photoreceptor development [13]. In animal studies with mice, *ATOH7* expression has been found in the developing optic nerve during embryogenesis [14]. During retinogenesis, seven different major classes of cells develop out of the progenitor cells in the eye: photoreceptors (rods and cones), bipolar cells, horizontal cells, amacrine cells, retinal ganglion cells (RGC; these are the cells involved in OAG) and Müller cells. Degeneration of these cells may lead to blindness [15]. In mutant mice and zebrafish without *ATOH7*, optic nerves and RGC are not further developed, while amacrine cells and cones are formed in excess [16], [17]. Overexpression of *ATOH7* and interaction with the *neuroD* gene in chickens increases the amount of RGC and photoreceptors [18]. The duration of expression of *ATOH7* is regulated by several proteins, including Growth and Differentiation Factor 11 (*GDF11*) [19]. Another factor involved in this genetic pathway is Sonic hedgehog (*SHH*), which mediates the direction of growth as the eye develops from the central part towards the periphery (including the optic nerve) [20]. Thus the *SHH* and *GDF11* regulate *ATOH7*, which in turn regulates *Brn3b*. This gene may play a role in further differentiation of RGC and is expressed in post-mitotic RGC precursors. First, RGC differentiate into the lower retinal epithelium (later becoming the RGC layer). At the same time, the dendrites reach the bipolar, horizontal, and amacrine cells in the inner retinal plexiform layer, while their axons form the optic nerve, optic chiasm, superior colliculus and lateral geniculate nucleus [20]. Although *ATOH7* has been implicated in retinal development in animals, this gene has not been linked to the development of the optic nerve pathology in humans. The analysis of VCDR showed that the *ATOH7* (rs1900004) was also significantly associated with VCDR, independent of optic disc area. This suggests that this gene is involved in both the optic disc area as in VCDR.

The 1p22 region is second in terms of strength of association based on the p-values. This region includes the genes *CDC7* and *TGFBR3* associated with optic disc area. *CDC7* encodes for a cell division cycle protein with kinase activity. Overexpression of this gene has been found in neoplastic transformations in some tumors. Although this region is associated with the optic disc area, the protein that *CDC7* encodes for interacts with the *CDKN2A* protein associated with VCDR. However, also the *TGFBR3* is of interest because of the interaction of *ATOH7* with *GDF11*, a member of the bone morphogenetic protein (BMP) and the TGFbeta superfamily. The genes therefore point to the same signaling pathway. *GDF11* interacts with the latent transforming growth factor beta binding protein 3 (*LTBP3*). In our analyses targeting VCDR, we found genome-wide significant evidence for a relation of *LTBP3* to VCDR (see below). While *CDKN2A* is not known to be involved in TGFbeta signaling, *CDKN2B* has been implicated in this pathway. As in the VCDR analysis, the most significant SNPs on chromosome 9p21 were located within the *CDKN2B* gene. This gene (also known as *p15Ink4b*) lies adjacent to the tumor suppressor gene *CDKN2A* and encodes a cyclin-dependent kinase. The protein encoded by *CDKN2B* is thought to play a role in cell growth regulation and is induced by transforming growth factor beta (*TGFB*) [21]. The *p15ink4b* protein phosphorylates and inactivates the retinoblastoma tumor suppressor (*pRb*) protein [22]. Deletions of this gene and of the retinoblastoma 1 gene are often found in malignant gliomas and melanomas [23]. A recent study in mice found that *p15Ink4b* was ectopically expressed in both zinc finger E-box binding homeobox 1(*Zeb1*) mutant cells and neuroectodermally derived cells, including the developing retina, optic nerve and muscles surrounding the eye [24]. Taken together, our findings point to a central role of TGFbeta in the development of the optic disc and VCDR. TGFbeta is a multifunctional cytokine that modulates developmental and repair processes in several tissues. TGFbeta signaling has been implicated in a wide variety of diseases including inflammation, autoimmune disorders, fibrosis, cancer and cataracts. The region has recently also been associated with myocardial infarction and type 2 diabetes mellitus [25]. The *CDKN2B/CDKN2A* and *CDC7*/*TGFBR3* loci influence the VCDR independently of optic disc area as these genes were not significantly associated with the optic disc area (p>0.05). However, *TGFBR3* appears to be involved in VCDR through its role in optic disc area, as the effect of this gene on VCDR increased two fold when we did not adjust for optic disc area (RS-I: unadjusted beta = 0.015, standard error = 0.004, p = 2.45×10^−5^ compared to beta = 0.007, standard error = 0.003 in the adjusted analysis).

Regarding the optic disc area, we found one additional region genome-wide significantly associated when pooling the data of the Dutch and TwinUK. Although the chromosome 16q12.1 region concerns a gene desert, the closest gene in the third locus associated with optic disc area is *SALL1*. Defects in this gene are a cause of Townes-Brocks syndrome and the bronchio-oto-renal syndrome, two autosomal dominant disorders [26]. Only rare variants have been implicated in Townes-Brocks syndrome and bronchio-oto-renal syndrome, while the association we report here is with common variants. One of the traits involved in the latter syndrome is myopia [27]. However, in our analyses we could not find evidence for an association of the common SNPs in the *SALL1* region to myopia (rs1362756; p = 0.802). *SALL1* encodes a zinc finger transcriptional repressor. When considering the protein pathway, *SALL1* interacts with *SIX1* [28]. Rare variants in *SIX1* are involved in the bronchio-oto-renal syndrome [29]. We found that common variants in *SIX1* were genome-wide significantly associated with VCDR.

Regarding VCDR, chromosome 14q22-23 was genome-wide significant in the discovery cohorts and was replicated consistently in the other cohorts. The region includes two genes which are obvious candidates *SIX1* and *SIX6* (the latter also known as *Optx2* and about 94kb distance from rs10483727). This gene is involved in eye development and has been related to congenital glaucoma. Defects in this gene have been associated with anophtalmia in mice [30] and in humans [31], [32]. Embryological studies have shown expression in the ventral optic stalk, which later becomes the optic nerve [33]. In the adult mouse retina, *Optx2* mRNA has been found in cells within the ganglion cell layer and inner nuclear layer [34]. This gene is expressed in the developing retina, optic nerve and other brain structures [31].

There were three more genome-wide significant loci on chromosomes 11q13, 13q13 and 22q12.1 associated with VCDR (Table 2). On 11q13 most SNPs were found close to *SCYL1*, which has been associated with optic nerve atrophy in mice [35]. However, also the presence *LTBP3* in this region is of interest, as this protein binds to *TGFB1*, *TGFB2*, and *TGFB3*, and is thus involved in the same signalling pathway as *CDKN2B*. *LTBP3* is further of interest because of its homology to *LTBP2*, which has been implicated in primary congenital glaucoma [36], [37]. The *DCLK1* gene on 13q13 is expressed in the optic tectum [38]. This is a probable kinase that may be involved in a calcium signaling pathway controlling neuronal migration in the developing and mature brain. Finally, the *CHEK2* gene has been associated with several types of cancer, including breast cancer [39]. A literature search did not show a direct link between *CHEK2* and the eye, however one study reported mapping of a locus on chromosome 22q12.1–q13.1 (*OPA5*) to autosomal dominant optic atrophy [40] and one case-report described an association of chromosome 22q11.2 deletion syndrome with optic disc swelling, which is probably caused by the resulting hypocalcaemia [41]. Regarding the association of *CHEK2* with breast cancer, it is of interest that also one borderline significant SNP is located in a gene breast carcinoma amplified sequence 3 (*BCAS3*) involved in this pathway.

Although our study has convincingly identified SNPs involved in optic disc area and VCDR, there are also a number of limitations. At this point, we cannot pinpoint the two endophenotypes to a single clinical outcome. There was some marginal evidence suggesting that four of the genes involved in the development of the optic disc area and VCDR are relevant for OAG. However, the findings were far from genome-wide significance and remain to be confirmed. Another limitation concerns the differences in methodology. Two of the four replication cohorts, RS-III and ERF, used confocal scanning laser ophthalmoscopy to determine the optic disc area, while the other studies, RS-I, RS-II and TwinsUK, used digitized stereoscopic images. Although this may be considered a drawback, we do not think this distorted our results, since, several studies compared both methods and found high correlations for all stereometric parameters [42]–[44]. Moreover, since our findings replicated in all cohorts differences across measurements are probably small and unlikely to influence our results, beyond that the estimation of the effects (beta-coefficients) may differ across studies. Finally, the TwinsUK study served as a replication cohort in this study, but is also involved as a replication cohort for a GWAS based on a discovery cohort from Australia (Macgregor, et al. unpublished data). Both, Dutch and Australian cohorts independently implicated *ATOH7* as playing a role in optic disc phenotypes and both utilize the TwinsUK data to replicate their findings. Although the association of *ATOH7* was genome-wide significant in the Dutch validation cohorts, this overlap in replication samples should be taken into account.

In conclusion, by conducting GWA analyses, we found genome-wide significant evidence for the association of three genetic loci associated with optic disc area, and another six with VCDR. Although multiple genes were included in the regions of interest, the most interesting ones for optic disc area were *TGFBR3* on chromosome 1p22, *ATOH7* on chromosome 10q21.3-22.1 (also for VCDR) and *SALL1* on chromosome 16q12. Regions of interest for VCDR were *CDKN2B* on chromosome 9p21, *SIX1* on chromosome 14q22-23, *SCYL1* on chromosome 11q13, *CHEK2* on chromosome 22q12.1, *DCLK1* on chromosome 13q13, and *BCAS3* on chromosome 17q23. There are several pathways implicated but the most interesting is the TGFbeta signaling pathway that appears to play a key role. Further research is needed to implicate these finding to pathology of the eye.

## Materials and Methods

### Study populations

The Rotterdam Study I (RS-I) is a prospective population-based cohort study of 7,983 residents aged 55 years and older living in Ommoord, a suburb of Rotterdam, the Netherlands [45]. Baseline examinations for the ophthalmic part took place between 1991 and 1993; follow-up examinations were performed from 1997 to 1999 and from 2002 to 2006.

The RS-II and RS-III are two other prospective population-based cohort studies of 3,011 residents aged 55 years and older and 3,392 residents aged 45 years and older respectively. The rationale and study design are similar to those of the RS-I [45]. The baseline examinations of RS-II took place between 2000 and 2002; follow-up examinations were performed from 2004 to 2005. Baseline examinations of RS-III took place between 2006 and 2009.

The Erasmus Rucphen Family (ERF) Study is a family-based cohort in a genetically isolated population in the southwest of the Netherlands with over 3,000 participants aged between 18 and 86 years. Cross-sectional examination took place between 2002 and 2005. The rationale and study design of this study have been described elsewhere [46], [47]. All measurements in these studies were conducted after the Medical Ethics Committee of the Erasmus University had approved the study protocols and all participants had given a written informed consent in accordance with the Declaration of Helsinki.

Finally, the TwinsUK adult twin registry is a volunteer cohort of over 10,000 healthy twins based at St Thomas' Hospital in London. Participants were recruited and examined between 1998 and 2008. A total of 843 had complete data, all of whom were Caucasian. This cohort is predominantly female, as only 3% of included participants were male.

### Ophthalmic examination

The ophthalmic assessment in RS-I and RS-II, both for baseline and follow-up, included a medical history, autorefraction, keratometry, visual field testing and optic nerve head imaging with Topcon ImageNet System of both eyes after mydriasis with topical tropicamide 0.5% and phenylephrine 2.5%. RS-III was similar to RS-I except for optic nerve head imaging with confocal scanning laser ophthalmoscopy (Heidelberg Retina Tomograph 2 [HRT]). The ophthalmic assessment in ERF included a medical history, autorefraction, keratometry and optic nerve head imaging with HRT of both eyes after pharmacologic mydriasis. In the TwinsUK optic disc parameters were measured from stereo disc photographs using the Nidek-3DX stereo camera, with digitized images scanned from Polaroid images and StereoDx stereoscopic planimetric software (StereoDx) using a Z-screen (StereoGraphics Corp) and software obtained from James Morgan from Cardiff University software, Wales, UK [48].

### Optic nerve head assessment

ImageNet, which was used in RS-I and RS-II, takes simultaneous stereoscopic images of the optic disc at a fixed angle of 20°, using a simultaneous stereoscopic fundus camera (Topcon TRC-SS2; Tokyo Optical Co., Tokyo, Japan). Images were analyzed using the ImageNet retinal nerve fiber layer height module. On each stereoscopic pair of optic disc images four points were marked on the disc margin, defined as the inner border of the peripapillary ring or the outer border of the neural rim, if a scleral ring was visible. Next, the software drew an ellipse using these points to outline the disc margin and to determine the cup. The amount of correspondence between the marked points on the two images of the stereoscopic pair is expressed as a “bad points” percentage, which indicates the percentage of points lacking correspondence. This percentage can be used as an indicator of image quality. Images with 25% or more bad points were excluded [49].

HRT 2, used in RS-III and ERF, uses a focused 670-nm diode laser light beam to acquire scans of the optic nerve head region, using the confocal principle. The HRT obtains, during one scan, three series of 16 to 64 confocal frontal slices. From each of these series, a 3-dimensional image of the optic nerve head is reconstructed, from which the software calculates several optic disc parameters. To define the cup, the HRT places a reference plane 50 mm below the peripapillary retinal surface in the region of the papillomacular bundle.

Imaging was performed after entering the participant's keratometry data into the software and after adjusting the settings in accordance with the refractive error. In RS-III all HRT 2 data was converted to HRT 3. As an indicator of image quality we used the topographic standard deviation of the scan, which is a measure of the variability among the three series of a single HRT scan. Scans with a topographic standard deviation exceeding 50 mm were excluded. The inter-observer variability and agreement for both systems have been described elsewhere [44]. Details of the optic disc measurements in TwinsUK are described elsewhere [50].

### Myopia and open-angle glaucoma assessment

Myopia was defined as a spherical equivalent of −6.00D or lower. For each eye the spherical equivalent was calculated using the standard formula: spherical equivalent = spherical component+(cylindrical value/2). The mean spherical equivalent of both eyes was included. Those eyes with a history of cataract surgery were excluded from this analysis.

OAG diagnosis was primarily based on glaucomatous visual field loss (VFL). The visual field of each eye was screened with a Humphrey Field Analyzer (HFA II 740; Zeiss, Oberkochen, Germany) using a 52-point threshold-related supra-threshold test that covered the central field with a radius of 24°. This test was modified from a standard 76-point screening test [51], [52]. VFL was defined as non-response in at least three contiguous test points (or four including the blind spot). If the first test was unreliable (>33% false-positive or false-negative catch trials) or a reliable test showed VFL in at least one eye, a second supra-threshold test was performed on that eye. If the second supra-threshold test was reliable and showed VFL, a full-threshold HFA 24-2 test (second follow-up) or Goldmann perimetry (Haag Streit, Bern, Switzerland; baseline and first follow-up) was performed on both eyes. The classification process of the Goldmann perimetry test results [51] and the full-threshold HFA 24-2 test results [Czudowska, et al. unpublished data] have been described before. In short, VFL was considered to be glaucomatous VFL only if reproducible and after excluding all other possible causes. For the present study, participants were considered as having glaucomatous VFL if they had glaucomatous VFL in at least one eye during either follow-up round. Cases had to have an open anterior chamber angle and no history or signs of angle closure or secondary glaucoma were allowed [52]. Criteria for glaucomatous optic neuropathy, such as VCDR, were not included in the criteria for OAG.

### Genotyping

In the RS-I, RS-II and RS-III cohorts, DNA was genotyped by using the Illumina Infinium II HumanHap550chip v3.0 array according to the manufacturer's protocols. Details are described elsewhere [53]. After exclusion of participants for reasons of low-quality DNA, a total of 5,974 participants were available with genotyping data from RS-I, 2,157 participants from RS-II and 2,082 from RS-III. In ERF, DNA was genotyped on four different platforms (Illumina 6k, Illumina 318K, Illumina 370K and Affymetrix 250K), which were then merged. After exclusion of participants for whom genotyping data were unavailable, 2,385 had genotyping data. As we did not use the same microarray for the various study populations we imputed our genotype data using HapMap CEU as reference population, resulting in over 2.5 million SNPs. Extensive quality control analyses have been performed in each cohort. Finally, the genotyping of the TwinsUK cohort took place in stages; in the first stage participants were genotyped by using Illumina's HumanHap 300K duo chip, whereas in the second stage participants were genotyped with Illumina's HumanHap610 Quad.

### Statistical analysis

#### Statistical analysis within studies

If we had data on both eyes then we chose a random eye. In cases of missing or unreliable baseline data on both eyes, we used follow-up data where available. Results from the RS-I and RS-II cohorts were combined, because both studies were identical in population structure. Within each study, linear regression models were used to examine the associations between SNPs and optic disc area adjusted for age and gender. The analyses of VCDR were further adjusted for optic disc area. Using these linear regression models, we calculated regression coefficients with corresponding 95% confidence intervals (CI). To adjust for multiple testing a p-value of 5×10^−8^ or less was considered statistically significant. As a secondary analysis we performed the analyses of VCDR with the same additive models but with further adjustment for intraocular pressure and its treatment.

All statistical analyses were performed using SPSS version 15.0.0 for Windows (SPSS inc., Chicago, IL, USA; 2006), MACH2 QTL as implemented in GRIMP [54] and R statistical package version 2.8.1 for Linux (www.r-project.org). For the analysis of the family based data we used the GenABEL package to adjust for relationships [55].

#### Meta-analysis

First, we replicated the top SNPs of the discovery cohorts in the two Dutch replication cohorts (RS-III and ERF). To adjust for familial relationships of participants in ERF we used the score test described by Chen and Abecasis which is implemented in the GenABEL package [56]. Meta-analyses were performed with Metal for Linux (www.sph.umich.edu/csg/abecasis/metal) to summarize the global effect through the four cohorts. To obtain optimal and unbiased results we used genomic control and the inverse variance method of each effect size estimate [57]. This was only done for the SNPs that were genotyped or imputed in all four cohorts. SNPs which deviated significantly from Hardy-Weinberg equilibrium (p<0.0001) or if they had a minor allele frequency <0.05 were excluded in the present study. Next, we replicated all top SNPs from the joint analysis of the four Dutch cohorts in a combined analysis with the TwinsUK.

Finally, we tested in RS-I whether the identified loci were associated with other ophthalmic traits such as myopia by using the spherical equivalent of the refractive error, and OAG based on optic nerve head appearance and glaucomatous visual field loss. This was done by using logistic regression analyses adjusted for age and gender.

## Supporting Information

Figure S1 Optic disc area Q-Q plots for the observed versus expected p-values for the discovery cohorts (A), the individual replication cohorts (B,C), and for the meta-analysis (D).
(0.20 MB TIF)

Figure S2 Vertical cup-disc ratio Q-Q plots for the observed versus expected p-values for the discovery cohorts (A), the individual replication cohorts (B,C), and for the meta-analysis (D).
(0.19 MB TIF)

Table S1 Characteristics of the open-angle glaucoma patients presented as mean ± standard deviation (range) unless stated otherwise.
(0.04 MB DOC)
