# Supplementary figures and images for: A Genome-Wide Association Study of Optic Disc Parameters
Source: PLoS Genet. 2010 Jun 10;6(6):e1000978. doi: 10.1371/journal.pgen.1000978 (PMC2883590; doi:10.1371/journal.pgen.1000978)

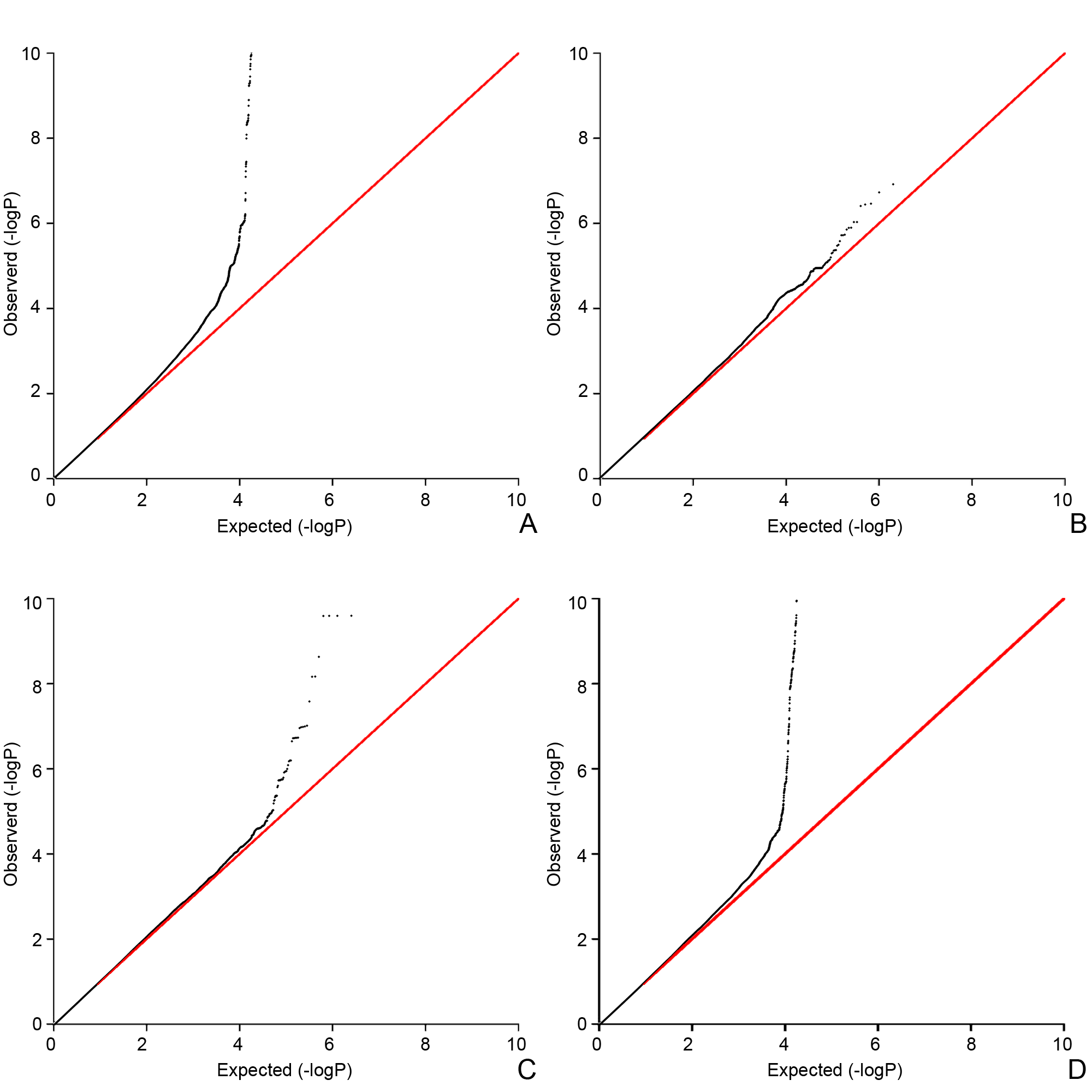

Supplement: Figure S1 — Optic disc area Q-Q plots for the observed versus expected p-values for the discovery cohorts (A), the individual replication cohorts (B,C), and for the meta-analysis (D). (0.20 MB TIF) [file pgen.1000978.s001.tif]

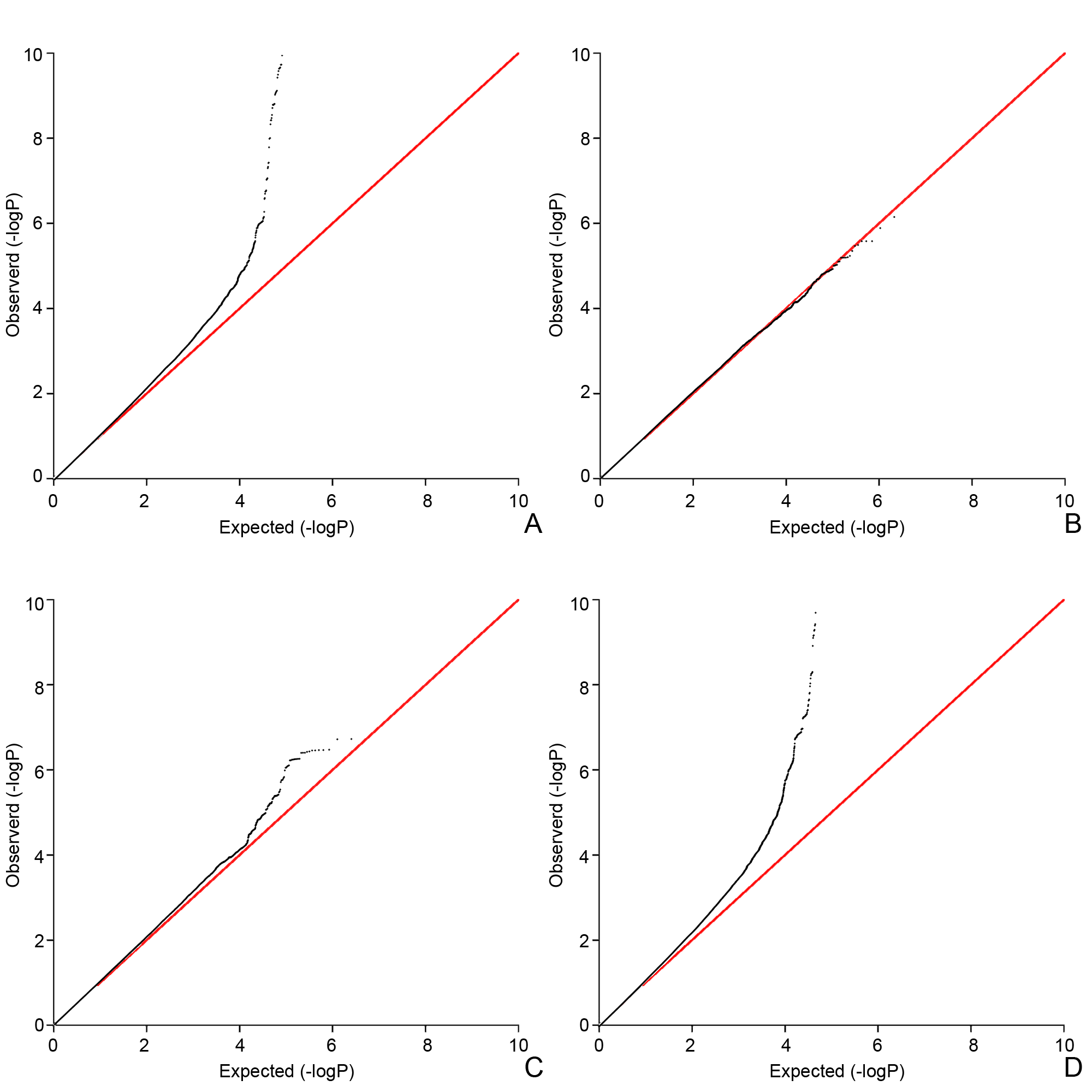

Supplement: Figure S2 — Vertical cup-disc ratio Q-Q plots for the observed versus expected p-values for the discovery cohorts (A), the individual replication cohorts (B,C), and for the meta-analysis (D). (0.19 MB TIF) [file pgen.1000978.s002.tif]
