# Supplementary material for: A Genome-Wide Association Study of Optic Disc Parameters
Source: PLoS Genet. 2010 Jun 10;6(6):e1000978. doi: 10.1371/journal.pgen.1000978 (PMC2883590; doi:10.1371/journal.pgen.1000978)
Supplement: Table S1 — Characteristics of the open-angle glaucoma patients presented as mean ± standard deviation (range) unless stated otherwise. (0.04 MB DOC) [file pgen.1000978.s003.doc]

Table S1. Characteristics of the open-angle glaucoma patients presented as mean ± standard deviation (range) unless stated otherwise

|  | RS-I |  |
| --- | --- | --- |
|  | Cases (N=188) | Controls (N=5,548) |
| Age (years) | 75.5 ± 7.4  (56 - 94) | 74.5 ± 7.8  (55 - 105) |
| Gender, N (%) female | 85 (45.2) | 3289 (59.3) |
| Intraocular pressure (mmHg) | 18.2 ± 6.2  (6.0 – 54.6) | 15.2 ± 3.5  (5.0 – 58.5) |
| Intraocular pressure treatment, N (%) | 37 (19.7) | 93 (1.7) |

RS = Rotterdam Study
